# Exercise Improves Lung Inflammation, but Not Lung Remodeling and Mechanics in a Model of Bleomycin-Induced Lung Fibrosis

**DOI:** 10.1155/2020/4302608

**Published:** 2020-10-16

**Authors:** Elias El-Mafarjeh, Gisele Henrique Cardoso Martins, Jessica Jorge Probst, Alana Santos-Dias, Manoel Carneiro Oliveira-Junior, Marcelo Paes de Barros, Luis Vicente Franco de Oliveira, Leandro Damas de Andrade, Renata Kelly da Palma, Renilson Moraes-Ferreira, Deborah de Camargo Hizume-Kunzler, André Luis Lacerda Bachi, Rodolfo P. Vieira

**Affiliations:** ^1^Department of Paediatrics, Sirio Libanes Hospital, Avenida Brasil 915, São Paulo SP, Brazil 01431-000; ^2^Brazilian Institute of Teaching and Research in Pulmonary and Exercise Immunology (IBEPIPE), Rua Pedro Ernesto 240, São José dos Campos SP, Brazil 12245-520; ^3^Department of Physical Therapy (LaPEx), University of State of Santa Catarina (UDESC), Avenida Madre Benvenuta 2007, Florianópolis SC, Brazil 88035-901; ^4^Interdisciplinary Postgraduate Program in Health Sciences, Institute of Physical Activity Sciences and Sports, Cruzeiro do Sul University, Rua Galvão Bueno 868, São Paulo SP, Brazil 01506-000; ^5^Post-graduation Program in Human Movement and Rehabilitation, Centro Universitário UniEvangélica, Avenida Universitária Km 3, 5, Anápolis GO, Brazil 75083-515; ^6^Department of Surgery, School of Veterinary Medicine and Animal Science University of São Paulo, São Paulo, Spain; ^7^Post-graduation Program in Bioengineering and Biomedical Engineering, Universidade Brasil, Rua Carolina Fonseca 235, São Paulo SP, Brazil 08230-030.; ^8^Post-graduation Program in Sciences of Human Movement and Rehabilitation, Federal University of São Paulo (UNIFESP), Avenida Ana Costa 95, Santos SP, Brazil 11060-001; ^9^Department of Otorrhynolaryngology, Federal University of São Paulo (UNIFESP), Rua Pedro de Toledo, 950-Vila Clementino, São Paulo SP, Brazil 04038-002; ^10^Post-graduation Program in Health Sciences, Santo Amaro University, Rua Isabel Schmidt, 349-Santo Amaro, São Paulo SP, Brazil 04743-030; ^11^Institute for Bioengineering of Catalonia, Biomimetic Systems for Cell Engineering (IBEC) C. Baldiri Reixac, 15-21 Barcelona, Spain 08028

## Abstract

**Introduction:**

Moderate aerobic exercise training accelerates the resolution of lung fibrosis in a model of bleomycin-induced pulmonary fibrosis. However, whether it can inhibit the development of lung fibrosis is unknown.

**Materials and Methods:**

C57Bl/6 mice were distributed into four groups: Control (Co), Exercise (Exe), Bleomycin (Bleo), and Bleomycin+Exercise (Bleo+Exe). A single bleomycin dose (1.5 UI/kg) was administered orotracheally and treadmill exercise started in the same day, enduring for 4 weeks, 5x/week, 60 minutes/session, at moderate intensity. Lung mechanics, systemic and pulmonary inflammation, and lung remodeling were evaluated. Lung homogenates were used to evaluate the antioxidant status.

**Results:**

Total cells, macrophages, lymphocytes, and neutrophils numbers, in agreement with IL-6 levels, were higher in the BAL and serum of Bleo group, compared to other groups. In addition, lung levels of LTB4 in Bleo were higher than other groups, whereas SOD activity and nitric oxide levels in exercised groups (Exe and Exe+Bleo) compared to the Bleo group. Lung GPX activity was lower in Bleo and Exe+Bleo groups compared to others. Exe and Exe+Bleo groups also showed higher IL-10 expression by lung macrophages than other groups, whereas TGF-*β* expression was higher in Exe, Bleo, and Exe+Bleo groups compared to control. CCR7 expression was induced only in the Exe group. However, exercise did not improve lung remodeling and mechanics, or serum and pulmonary levels of VEGF, IGF-1, and TGF-*β*.

**Conclusion:**

Aerobic exercise training initiated concomitantly with induction of pulmonary fibrosis reduces lung and systemic inflammation but fails to inhibit lung fibrosis and mechanics impairment.

## 1. Introduction

Idiopathic pulmonary fibrosis (IPF) is a devastating disease, leading to death normally between 3 and 5 years after the diagnostic [1]. The main symptoms include dyspnea and cough, reflecting the rapidly pulmonary remodeling, decrease of lung function, and subsequent permanent hypoxemia [2]. In addition, there is no curative treatment for IPF, and many studies emerged to ensure the survival and improve the quality of life of these patients, by preserving lung function and minimizing adverse effects of therapy [1–3]. Accordingly, many other clinical studies have also been conducted to evaluate the effects of pulmonary rehabilitation programs for IPF patients [4–7]. Monitored physical exercise in individuals with IPF reduces dyspnea incidence, improves exercise performance (distance covered, effort tolerance, and aerobic capacity), and brings many health benefits, such as higher quality of life and lower symptoms of disease [4–7]. However, there is scarce information on the mechanisms by which exercise ameliorates IPF condition, since only few preclinical studies shed some light in the cellular and molecular events associated [8, 9].

Recently, our group (and others) showed that moderate intensity aerobic exercise training can accelerate the resolution of bleomycin-induced pulmonary fibrosis [8, 9]. However, one question remains unsolved: could exercise inhibit the development of fibrosis if the training program begins concomitant with the disease initiation?

Thus, the present study investigated the antioxidant and inflammatory and fibrotic lung responses of moderate aerobic exercise initiated concomitantly with bleomycin administration, thereby modeling an early nonpharmacological intervention after the diagnosis of IPF.

## 2. Material and Methods

All experimental procedures were approved by the ethical committee from School of Medicine of University of Sao Paulo (375/13). These procedures were carried out in accordance to Guide for the Care and Use of Laboratory Animals, published by the U.S. National Institutes of Health.

### 2.1. Animals and Experimental Groups

Three sets of six C57Bl/6 mice (total of 18 animals per group) were used for this study. Mice (20-25 g) were obtained from the Central Animal Facility of School of Medicine of the University of São Paulo and distributed equally in Control (Co), Exercise (Exe), Bleomycin (Bleo) and Exercise+Bleomycin (Exe+Bleo) groups.

### 2.2. Experimental Model of Pulmonary Fibrosis Induced by Bleomycin

Sulfate of bleomycin (1.5 UI/kg; Meizler Biopharma, SP, Brazil) was administered orotracheally under anesthesia (ketamine 100 mg/kg and xylazine 10 mg/kg) on day 1 of the experimental procedures, which corresponded to first day after the initial physical test (after the three days of adaptation). Bleomycin-induced lung fibrosis, when administered intratracheally at doses ranging between 1.25 UI/kg to 4 UI/kg, remains the best experimental model available until this moment [10].

### 2.3. Treadmill Exercise Test and Training

Treadmill exercise adaptation, test, and training were performed as previously described [11–14]. Briefly, after 3 days of adaptation (15 min/day, 25° incline, 0.2 km/h) on the treadmill, animals were submitted to a physical test (starting at 0.2 km/h and increasing speed by 0.1 km/h every 2.5 min until exhaustion, i.e., until animals were not able to run, even after 10 mechanical stimulus). Then, animals entered the exercise training program for 4 weeks, 5x/week, 60 min/session, 60% of maximal velocity reached in the preliminary physical test. Euthanasia was performed twenty-four hours after the last session of exercise training [11–14].

### 2.4. Blood and Bronchoalveolar Lavage (BAL) Collection and Analysis

Under anesthesia (ketamine (100 mg/kg) and xylazine (10 mg/kg)), 1 mL of blood was collected via vena cava, and serum was isolated (by centrifugation at 1000g, at 4°C, 10 min), collected, and stored at -80°C for cytokine measurements.

Following blood collection, mice were tracheostomized and cannulated for BAL collection. The lungs were washed with 1.5 mL of PBS, 1 mL was recovered, and then centrifuged at 900g for 10 min at 4°C. The supernatant was stored at -80°C for cytokines and growth factors measurements. The cell pellet was resuspended in 1 mL of PBS and the total cell number was calculated using a hemocytometer (Neubauer chamber), as well as the differential cell counting, performed by microscopic examination of Diff Quick-stained cytospin preparations (300 cells per slide) [9, 15–17].

### 2.5. Cytokines Measurements in BAL and Serum

The concentrations of VEGF, IL-6, IGF-1, and TGF-beta in serum were measured using ELISA kits, according to the manufactures' recommendations (R&D Systems, MN, USA) [8, 11–13].

### 2.6. Collagen Fibers Quantification in the Lung Parenchyma

Lungs were excised and separated in two parts. One part was paraffin-embedded and submitted to histological routine. Five micrometers of lung slices was stained with picrosirius red [16–18]. The content of collagen fibers (areas of the lung that stained red) in the parenchyma was quantified by image analysis using the Image Pro Plus 4.5 software as previously described [16–18]. The results were expressed as the percentage of collagen fibers related to the total amount of lung tissue.

### 2.7. Lung Homogenate Preparation

After lung excision, a part of tissue was mixed with 5 mL of phosphate-saline buffer (PBS 1×, pH 7.3) and macerated. The solution obtained was centrifuged (4 min at 1400g), and 1 mL of the supernatant was stored at −80°C for further analysis of leukotriene B_4_ (LTB_4_) and nitric oxide (NO) concentrations, and enzyme activities of superoxide dismutase (SOD), glutathione reductase (GR), and glutathione peroxidase (GPX) [19].

### 2.8. Leukotriene and Nitric Oxide Determination in the Lung Homogenates

The concentration of LTB_4_ (MyBioSource, San Diego, CA) was determined in the lung homogenate by an ELISA kit, following the manufacturer's instructions (MyBioSource, San Diego, CA). Nitric oxide (NO) concentration in the lung homogenate was evaluated using the Griess reaction. Samples (50 *μ*L) were placed in triplicates in a microplate (96-well plate), and a volume of 50 *μ*L of Griess reagent (0.1% naphthyl ethylenediamine dihydrochloride, 1% sulphanilamide, and 2.5% orthophosphoric acid) was added. After 10 minutes of reaction at room temperature in the dark, the NO concentration was determined at absorbance of 570 nm using a microplate reader [19].

### 2.9. Determination of Antioxidant Enzyme Activities

The antioxidant enzymes SOD, GPX, and GR were assayed in reaction volumes of 250 *μ*L using a 96-well microplate spectrophotometry reader SpectraMax M5 (Molecular Devices, Sunnyvale, CA, USA). SOD activity was measured using the method described by Ewing and Janero (1995) which involves the reduction of superoxide radicals (O_2_^•−^) by nitro-blue tetrazolium (NBT), at 540 nm, following a linear first-order kinetic during 3 min. Microplate superoxide dismutase assay employing a nonenzymatic superoxide generator. One SOD unit (1 U_SOD_) represents the amount of enzyme necessary to inhibit 50% of the formazan production rate obtained in blank reaction (25°C, 1 atm). GPX and GR activities were measured based on the oxidation of *β*-NADPH, monitored by spectrophotometry at *λ* = 340 nm (molar absorption *ϵ*_340nm_ = 6.22x10^3^ M^−1^ s^−1^). GPX assay uses reduced glutathione (GSH) and *tert*-butyl hydroperoxide as substrates, whereas the GR assay uses oxidized glutathione (GSSG). Protein determination was performed by the Bradford protocol. A rapid and sensitive method for the quantitation of microgram quantities of protein utilizes the principle of protein-dye binding [19].

### 2.10. Flow Cytometry Study for Macrophage Activation and CCR7 Expression

The cells from BAL were counted using a Neubauer chamber and adjusted to 1 × 10^6^ cells per mL. The cells were stained with fluorescent-conjugated monoclonal antibodies for determination of the inflammatory cells CD3+ FITC (lymphocytes), CD11b PERCP (macrophages), Ly6G APC (granulocytes neutrophils and eosinophils), and CCR7 PE (chemokine receptor 7), all from (Becton Dickinson–BD™, East Rutherford, NJ, USA). The following cytokines (IL-10, IL-12, and TGF-beta (LAP); all in PE) in each cell type were also analyzed (Becton Dickinson–BD™, East Rutherford, NJ, USA), by using the surface marker with posterior intracellular marker using CitoFix/CitoPerm permeabilization kit (Becton Dickinson–BD™, East Rutherford, NJ, USA). The readings were done using the FACS Accury C6 (Becton Dickinson–BD™, East Rutherford, NJ, USA) [19].

### 2.11. Measurements of Lung Mechanics by Movement Equation and by Expiratory Pause

Briefly, mice were anesthetized with a ketamine (100 mg/kg) and xylazine (10 mg/kg), tracheostomized, and ventilated by using a volumetric ventilator (MV215, Montevideo, UY) in two different ways, open and closed chest [20]. The parameters of ventilation used were a quasi-sinusoidal flow pattern with a tidal volume of 10 ml/kg of mouse body weight, a frequency of 100 breaths/min, and a positive end expiratory pressure of 2 cm H_2_O. Flow and pressure signals from the transducers were analogically low-pass filtered, sampled, and stored for subsequent analysis. Using these parameters, the static (*E*_st_) and dynamic elastance (*E*_dyn_) were obtained and analyzed. The results were expressed in cm H_2_O ml^−1^ [19].

### 2.12. Statistical Analysis

The GraphPad Prism 5.0 software was used to perform the statistical analysis and to construct the graphs. Normality analysis revealed parametric data that were expressed as the mean ± SD using bar plus the standard error bar. Comparisons between all groups were carried out by a two-way analysis of variance (ANOVA), followed by the Student-Newman-Keuls post hoc test. Values were considered significant at *p* < 0.05.

## 3. Results

### 3.1. Exercise Training Reduces Bleomycin-Induced Lung Inflammation

**Figure 1:**
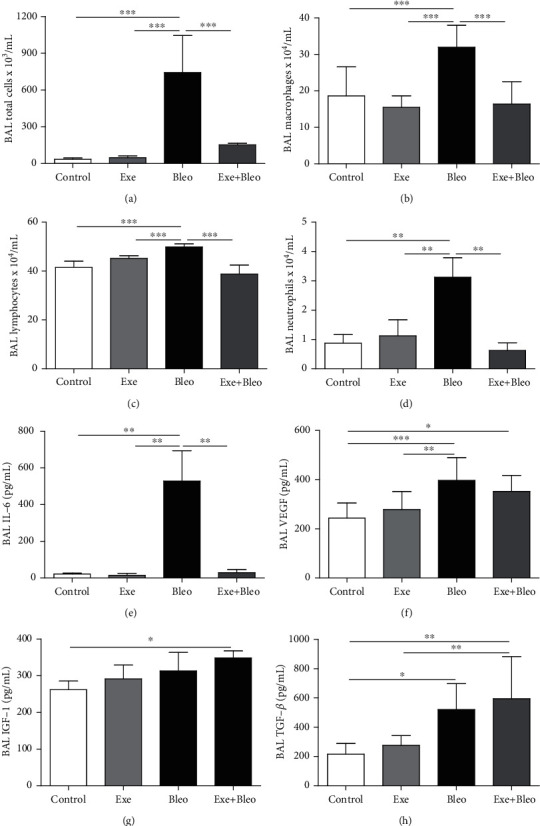
Inflammatory cell, cytokines, and growth factor measurements. Number of total cells (a), macrophages (b), lymphocytes (c), neutrophils (d), and levels of cytokines IL-6 (e), VEGF (f), IGF-1 (g), and TFG-*β* (h) in BAL fluid from Control (Co), Exercise (Exe), Bleomycin (Bleo), and Bleomycin+Exercise (Bleo+Exe) groups. ∗∗∗*p* < 0.001, ∗∗*p* < 0.01, and ∗*p* < 0.05.

As shown in Figure 1, a higher number of total cells (Figure 1(a), *p* < 0.001), macrophages (Figure 1(b), *p* < 0.001), lymphocytes (Figure 1(c), *p* < 0.001), and neutrophils (Figure 1(d), *p* < 0.01) were found in the BAL isolated from the Bleo group compared to other groups. IL-6 levels (Figure 1(e), *p* < 0.01) were also higher in the Bleo group compared to others. VEGF levels (Figure 1(f)) in the BAL of the Bleo group were significantly higher than Co (*p* < 0.001) and Exe (*p* < 0.01) groups, although VEGF levels in the Exe+Bleo group were only higher than Co (*p* < 0.05). Likewise, the IGF-1 content in BAL from the Exe+Bleo group was only higher than the Co group (Figure 1(g); *p* < 0.05). Moreover, the TGF-*β* content in the Bleo group was significantly higher than Co (*p* < 0.05), whereas the Exe+Bleo animals showed higher TGF-*β* levels than both the Co and Exe groups (Figure 1(h); *p* < 0.01). Finally, Figure 1(i) depicts higher levels of LTB4 in lung homogenates of Bleo animals compared with any other group (*p* < 0.001 versus Co and Exe; *p* < 0.01 versus Exe+Bleo).

### 3.2. Exercise Training Reduces Systemic Inflammation

**Figure 2:**
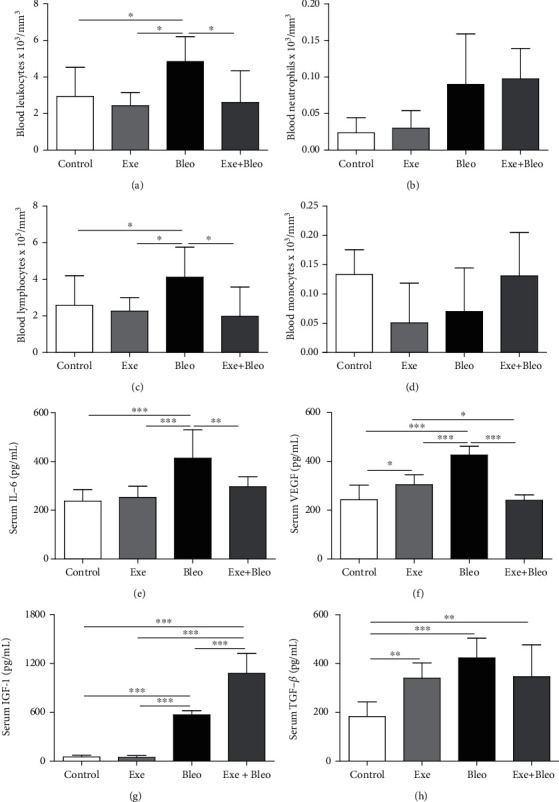
Inflammatory cell, cytokines, and growth factor measurements. Number of leukocytes (a), neutrophils (b), lymphocytes (c), monocytes (d), and the levels of cytokines IL-6 (e), VEGF (f), IGF-1 (g), and TFG-*β* (h) in serum from Control (Co), Exercise (Exe), Bleomycin (Bleo), and Bleomycin+Exercise (Bleo+Exe) groups. ∗∗∗*p* < 0.001, ∗∗*p* < 0.01, and ∗*p* < 0.05.

Figure 2 shows that the number of leucocytes (Figure 2(a), *p* < 0.05) and lymphocytes in blood (Figure 2(c), *p* < 0.05), but not of neutrophils (Figure 2(b)) and monocytes (Figure 2(d)), was higher in the Bleo group compared to other groups. In a similar way, serum IL-6 levels found in the Bleo group (Figure 2(e)) were increased compared to the Co (*p* < 0.001), Exe (*p* < 0.001), and Exe+Bleo group (*p* < 0.01). Regarding serum VEGF (Figure 2(f)), Bleo animals showed higher concentrations than other groups (*p* < 0.001), and VEGF content in the Exe group was only higher than the Co and Exe+Bleo groups (*p* < 0.05). IGF-1 levels in serum of Exe+Bleo animals were higher than other groups (*p* < 0.001), while Bleo samples showed increased levels only compared to Co and Exe groups (Figure 2(g); *p* < 0.001). Serum TGF-*β* levels (Figure 2(h)) were significantly increased in the Exe (*p* < 0.01), Bleo (*p* < 0.001), and Exe+Bleo (*p* < 0.01) groups compared to the Co group.

### 3.3. Exercise Training Modulated NO, SOD, and GPX Responses in Lung Tissue

**Figure 3:**
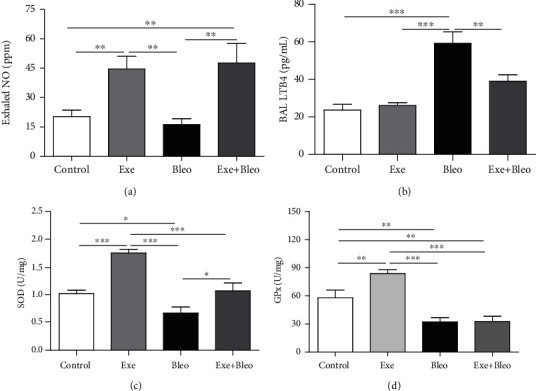
Levels of nitric oxide, leukotriene B4, and antioxidant proteins. Exhaled NO (a), LTB4 (b) in BAL fluid, superoxide dismutase (c), glutathione peroxidase (d) for Control (Co), Exercise (Exe), Bleomycin (Bleo), and Bleomycin+Exercise (Bleo+Exe) groups. ∗∗∗*p* < 0.001, ∗∗*p* < 0.01, and ∗*p* < 0.05.

Figure 3(a) depicts higher concentrations of exhaled NO in trained animals compared to untrained ones (*p* < 0.001). Corroborating with this observation, higher concentrations of NO were found in the lung homogenates from trained mice compared to control (Figure 3(b), *p* < 0.001). Regarding SOD activities (Figure 3(c)), the Bleo group presented lower SOD activities compared to other groups (*p* < 0.05 versus the Co and Exe groups; *p* < 0.001 versus the Exe group). In addition, the SOD activity of Exe animals was also higher than those found in the Co and Exe+Bleo groups (*p* < 0.001).  Figure 3(d) shows that GPX activities in the bleomycin-treated mice were lower than in other groups (*p* < 0.01 versus the Co group; *p* < 0.001 versus the Exe group), whereas GPX activity was higher in the Exe group compared to Co (*p* < 0.01). No significant changes were observed in GR activities between experimental groups (Figure 3(e)).

### 3.4. Exercise Training Modulates the Expression of IL-10, IL-12, TGF, and CCR7 in the Lung Macrophages

**Figure 4:**
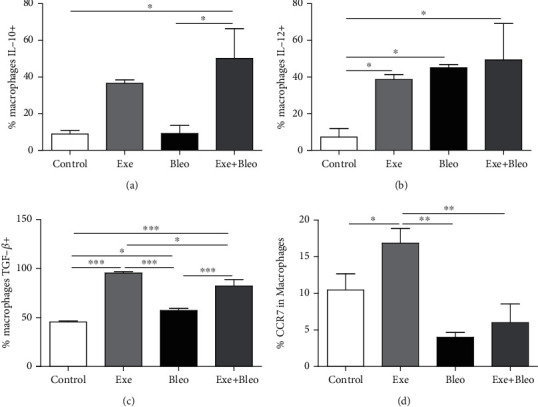
Cytokines in macrophage activation. Interleukin 10 (a), interleukin 12 (b), transforming growth factor *β* (c), and CCR7 (d) for Control (Co), Exercise (Exe), Bleomycin (Bleo), and Bleomycin+Exercise (Bleo+Exe) groups. ∗∗∗*p* < 0.001, ∗∗*p* < 0.01, and ∗*p* < 0.05.

As shown in Figure 4, IL-10 expression in lung macrophages from the Exe+Bleo group was increased by *x*% as compared to the Co and Bleo groups (Figure 4(a), *p* < 0.05). Concerning IL-12 expression (Figure 4(b)), Co group values were significantly lower than all other groups (*p* < 0.05). On the other hand, TGF-*β* expression in pulmonary macrophages from Exe mice (Figure 4(c)) was higher than that from other groups (*p* < 0.001 versus the Co and Bleo group; *p* < 0.05 versus the Exe+Bleo group). Higher values were also found for the Exe+Bleo group compared to the Co and Bleo groups (*p* < 0.001), as well as TGF-b expression in the Bleo group compared to Co (*p* < 0.05). The Exe group presented higher CCR7 expression values than all other groups (*p* < 0.001 versus the Bleo and Exe Bleo groups; *p* < 0.05 versus Co; Figure 4(d)).

### 3.5. Exercise Training Did Not Alter the Effect of Bleomycin in the Parenchymal Collagen and Lung Mechanics

**Figure 5:**
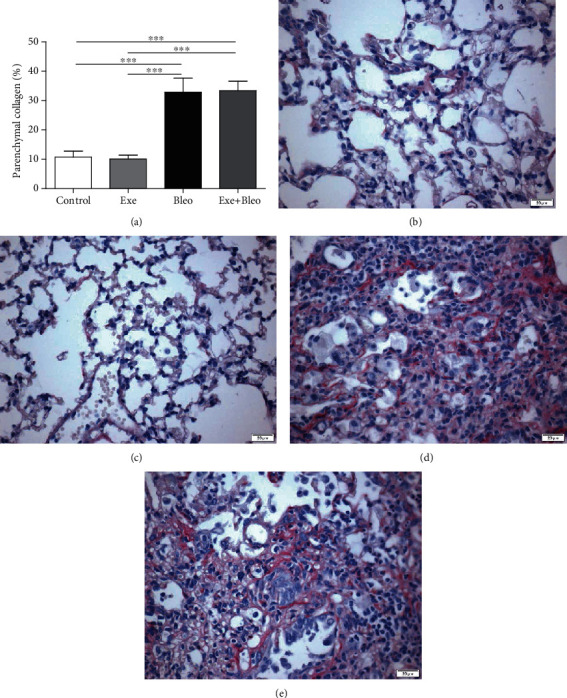
Effects of exercise training on collagen deposition in the lung parenchyma. % parenchymal collagen for all groups (a) and representative photomicrographs for (b) Control (Co), (c) Exercise (Exe), (d) Bleomycin (Bleo), and (e) Bleomycin+Exercise (Bleo+Exe) groups. ∗∗∗*p* < 0.001.

Figure 5 shows that the groups administered with bleomycin (Bleo and Exe+Bleo) showed increased parenchymal collagen content when compared to the Co and Exe groups (*p* < 0.001).

**Figure 6:**
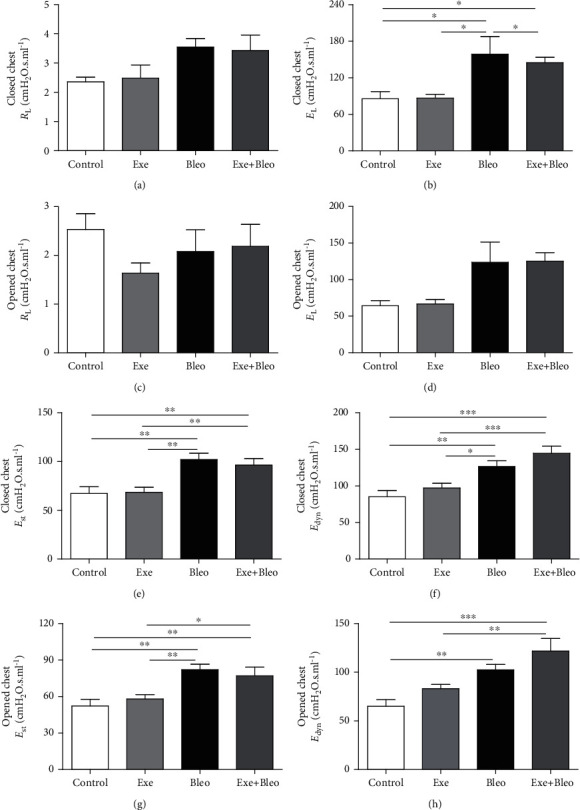
Effects of AT on the lung mechanics. Lung resistance (a, c), lung elastance (b, d), dynamic elastance (e, g), and staticelastance (f, h). Control (Co), Exercise (Exe), Bleomycin (Bleo), and Bleomycin+Exercise (Bleo+Exe) groups. ∗∗∗*p* < 0.001, ∗∗*p* < 0.01, and ∗*p* < 0.05.

Regarding pulmonary mechanics (Figure 6), there were no differences in pulmonary resistance (RL) both with closed (Figure 6(a)) and open chest (Figure 6(c)), whereas pulmonary elastance (EL) was higher in Bleo group compared to other groups (*p* < 0.05). Exe+Bleo animals also showed higher EL compared to Co (*p* < 0.05), when the chest was closed (Figure 6(b)). No differences in EL were found when the chest was open (Figure 6(d)). The analysis of elastic elasticity (*E*_st_) showed that the bleomycin-administered (Bleo) groups had increased *E*_st_ compared to Co and Exe groups, both with closed (Figure 6(e); *p* < 0.01) and open chest (Figure 6(g); *p* < 0.01 between and Co or Exe; *p* < 0.01 between the Exe+Bleo and Co groups; *p* < 0.05 between the Exe+Bleo and Exe groups). Data from dynamic elastance (*E*_dyn_) assessment showed that bleomycin-administered groups had increased *E*_dyn_ as compared to the Co and Exe groups, both with closed (Figure 6(f); *p* < 0.001 between Exe+Bleo and Co or Exe; *p* < 0.01 between the Bleo and Co groups; *p* < 0.05 between the Bleo and Exe groups) and open chest (Figure 6(h); *p* < 0.001 between the Exe+Bleo and Co or Exe groups; *p* < 0.01 between the Bleo and Exe groups).

## 4. Discussion

Our findings here demonstrate that, even though the exercise training was not able to alter the collagen deposition in the parenchyma or even some key pulmonary mechanic parameters, the aerobic exercise training putatively reduced the recruitment of immune system cells, the release of proinflammatory mediators/cytokines both in the lung and blood, and increased the IL-10 expression by lung macrophages of mice submitted to an experimental model of bleomycin-induced lung fibrosis. It is worth to mention that the findings of Pereira et al. [8] and Andrade-Souza et al. [21] corroborate our results, since these authors demonstrated that the exercise training promoted a significant reduction of immune cells number (and also proinflammatory cytokines) in the BAL of mice presenting pulmonary fibrosis compared to nonexercised control. However, the novelty of our study is settled on the benefits of the aerobic exercise training, here proposed as a “nonpharmacological intervention,” starting from the very beginning of lung aggression induced by bleomycin administration, while in the study from Pereira et al. [8] and Andrade-Souza [21], the exercise training started after two weeks of bleomycin administration.

Is it widely accepted that exercise training can ameliorate several pulmonary inflammatory symptoms [13–15], in a general way, due to the prominent anti-inflammatory effects associated. For instance, previous investigations on asthma models have identified that aerobic exercise training reduces the activity of leukotrienes and their mediators [22]. Leukotrienes are proactive inflammatory bioactive molecules, derived from the inflammatory lipoxygenase pathway, that act on the respiratory system as bronchoconstrictor, in the formation of edema, in mucus hypersecretion, and in the proliferation, activation, and survival of inflammatory cells [23, 24]. In the context of pulmonary fibrosis, it has been highlighted that 5-lipoxygenase (5-LO) enzyme and its main product, the leukotriene B4 (LTB4), have an important role in the development and pathophysiological aspects of this disease [25]. Corroborating this fact, Wilborn et al. (1996) demonstrated not only that the 5-LO expression in the alveolar macrophages of IPF patients was higher than control patients but also that the levels of LTB4 in the homogenates from IPF patients was 15-fold higher than that in control group, and such increased LTB4 levels were significantly correlated with inflammation and fibrosis lung indexes [26]. In addition, it was found that the LTB4 blockade by LTB4 receptor antagonist inhibited the bleomycin-induced pulmonary fibrosis [27]. It is also worth to mention that LTB4 acts as potent neutrophil chemoattractant and putative elevations of this molecule in the IPF could culminate in the accumulation of neutrophils in the lung. Based on that, the significant reduction of LTB4 levels in the lung homogenate and the neutrophil infiltration in exercise trained mice group with fibrosis observed here confirms the ability of aerobic exercise training to promote an anti-inflammatory status, even when the exercise training was initiated at the same day of lung fibrosis induction by bleomycin administration.

In this study, we can affirm that the aerobic exercise training was able to impose an pulmonary and systemic anti-inflammatory condition, based on the lower total leukocyte, macrophage, and lymphocyte numbers, as well as the IL-6 levels both in the lung and blood of trained fibrosis mice group (Bleo+Exe) compared to the nontrained mice group with fibrosis (Bleo). Similarly, Perreira et al. [8] and Andrade-Souza [21] verified a reduction in the total number of immune system cells and proinflammatory cytokines in the BAL of mice group with fibrosis submitted to exercise training compared to nontrained mice group with fibrosis. In addition, the same authors also reported an increase of IL-10 levels in BAL from trained mice group with fibrosis. By the way, findings from some studies have pointed that the elevations of the anti-inflammatory and immunosuppressive cytokines, such as IL-10, can attenuate the bleomycin-induced lung fibrosis [20, 28]. Therefore, it is tempting to suggest that our observation of higher IL-10 expression in lung macrophages of trained mice group with IPF could be directly involved in the reduction of lung inflammation, beyond to reinforce that physical exercise can positively activate lung macrophages, increasing IL-10 synthesis.

Macrophages respond to IL-10 and secrete anti-inflammatory mediators, such as TGF-*β*1, which can dampen the inflammation and preserve the tissue functions [29]. By our knowledge, it is the first time that higher expressions of TGF-*β* and IL-10 in pulmonary macrophages of trained mice group could act a major regulating factor of macrophage activation to the M2 profile, this, presenting a positive anti-inflammatory phenotype [29–32]. This deviation for an M2 profile was corroborated by the lower CCR7 expression in lung macrophages from trained mice group with fibrosis, since those macrophages with M1 profile increase the CCR7 expression, as evidenced in trained mice group without fibrosis [29–32].

Several studies reported that TGF-*β*1 and other growth factors, such as IGF-1 and VEGF, are actively involved in the development of pulmonary fibrosis [8, 33–35]. Although the exercise training was able to decrease the systemic VEGF levels in the trained mice group with IPF (Bleo+Exe) compared to nontrained mice group with IPF (Bleo), higher levels of TGF-*β*, IGF-1 and VEGF, both in serum and BAL of the IPF mice, can support our findings that the percentage of collagen deposition in the parenchyma and also the pulmonary mechanics parameters were unchanged in these experimental groups. These results are in opposite to our previous data [8, 21], in which a significant reduction of all these parameters was observed in exercised bleomycin-treated mice compared to nontrained fibrosis mice. Noteworthy, different from our previous studies, the exercise intervention here was initiated concomitantly to the bleomycin administration, which could reflect a distinguished physiological stimulus for pulmonary fibrosis induction remediation.

Nevertheless, in this study, the aerobic exercise training was not able to alter all the parameters aforementioned, but an important result found here was the increased nitric oxide (NO) levels in trained mice, both in exhaled air and lung homogenates. The role of NO in the pulmonary fibrosis pathology is still unclear and contradictory, since some studies mentioned that higher NO levels precede fibrotic changes in the lung or could mediate bleomycin-induced angiogenesis by VEGF regulation [36]. On the other hand, Noguchi et al. showed that NO could exert a protective effect in the bleomycin-induced pulmonary fibrosis [37]. Interestingly, the NO levels here were unaltered in nontrained fibrosis mice. Accordingly, Thornadtsson and collegues showed that higher NO levels in trained mice could reflect an adaptation of the lung to exercise training, which could lead to oxygen uptake improvement [38]. Therefore, we can suggest that aerobic exercise training could facilitate oxygen uptake through the NO-releasing in the pulmonary environment, even with alterations induced by bleomycin administration.

**Figure 7:**
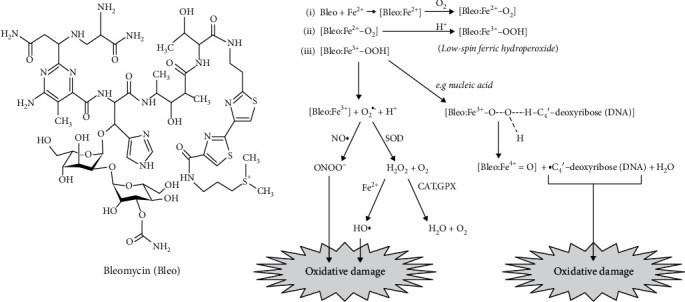
Role of [Bleo:Fe^2+^-OOH] complex in ROS/RNS signaling using desoxynucleic acid (DNA) as a central target.

Beyond the physiological role of NO• in exercise training, we cannot exclude the key participation of the vasodilator factor in the redox metabolism of lungs [39]. Bleo notoriously generates reactive oxygen and nitrogen species (ROS/RNS) by chelating redox-active ferrous ions (complex [Bleo:Fe^2+^]), then reacting with molecular oxygen (O_2_) to form the highly reactive low-spin ferric hydroperoxide [Bleo:Fe^2+^-OOH] [40]. The Bleo-ferric hydroperoxide spontaneously decomposes to produce the superoxide radical O_2_•-, which, *per se*, is not highly reactive with most vital biomolecules in cells. However, the O_2_•- radical is recognized as the main precursor of other aggressive ROS/RNS, such as peroxynitrite (formed by O_2_•- reaction with NO•, *k*_1_ ~ 6 − 9 × 10^9^ M^−1^ cm^−1^) [41] and, indirectly, the hydroxyl radical, HO•, which is actually formed by the reaction of hydrogen peroxide (H_2_O_2_, product of O_2_•- dismutation) with Fe^2+^ ions (the Fenton/Haber-Weiss reaction) [42]. Apart from its role in direct ROS/RNS generation, Bleo-ferric hydroperoxide is reactive enough to abstract a H• atom from a vicinal biomolecule—nucleic acids, unsaturated lipids, carbohydrate, and thiol- or tyrosine-dependent proteins—to initiate a free radical chain reaction that culminates in oxidative damage [43]. The participation of activated [Bleo:Fe^2+^-OOH] complex in ROS/RNS generation and inducer of oxidative damage is summarized in Figure 7, using desoxynucleic acid (DNA) as a potential biomolecule target [44].

On the other hand, recent findings have also shown that the extracellular redox environment is crucial in determining the extension of oxidative injuries in bleomycin-induced pulmonar fibrosis [45]. Extracellular sources of bleomycin-induced oxidative damage include enzymes such as NADPH oxidase, myeloperoxidase, and nitric oxide synthase-2 (Nox2), which are directly associated with BAL antioxidant depletion in pulmonar fibrosis [40]. In a common sense, the integration of extra- and intracellular antioxidant defenses is essential for efficient redox adjustments in order to prevent harmful oxidative damage in all cells exposed to adverse conditions [46]. Such a complicated temporal-spatial redox network is particularly executed by redox signaling molecules, such as H_2_O_2_, NO•, and NO_2_-tyrosine peptides, through redox-switch thiol-proteins (like GSH, thioredoxins, and peroxiredoxins) linked to antioxidant gene-responsive pathways, such as Keap1-Nrf2 and NF-*κ*B [47, 48]. Interestingly, regular moderate exercises are reported to increase ROS/RNS production (although at controlled levels) sufficient to trigger Keap1-NRf2 and NF-*κ*B pathways and induce adaptive antioxidant responses that improve several physiological functions (endocrine, cardiovascular, cognitive, etc.) for general health benefits [47–49].. In addition, it has been highlighted that the NF-*κ*B, a nuclear transcription factor, is a prominent mediator of several inflammatory diseases and its inhibition can prevent or even mitigate the inflammation-related pulmonary illness [50].. In this regard, it was reported that regular exercise training was able to reduce the NF-*κ*B expression, which led to the reduction of the levels of TNF-a and IL-6, in mice submitted to an experimental model of lung inflammation induced by water pipe smoke [47]. Although we did not investigate the activation of Nrf2 or NF-*κ*B signaling pathways here, it is tempting to suggest that the moderate exercise program applied for our experimental (Exe) and (Exe+Bleo) groups resulted in an upgraded antioxidant capacity in the pulmonary tissues of these animals, concomitant with Bleo administration, which offered higher defensive capacity of their lungs against the oxidative injury imposed by Bleo treatment. Further studies are necessary to address the putative role of both Nrf2 and NF-*κ*B signaling pathways in attenuating Bleo-linked oxidative injuries in pulmonary fibrosis models.

Beyond the elevation of NO levels in the trained mice groups, we also observed increased SOD activity in the same groups compared to the mice group with fibrosis. Reduction of SOD activity is a result widely verified in the bleomycin-induced pulmonary fibrosis, due to the bleomycin toxicity expressed as oxidative damage in lungs caused by overproduction of reactive oxygen species (ROS) [50–52]. The oxidative stress in lung tissues is strongly associated with depletion of antioxidant levels (e.g., glutathione and GSH), although counteractions from responsive antioxidant enzymes, such as superoxide dismutase and catalase, are often observed [50–52]. Corroborating our results, it has been documented that exercise training is able to improve the antioxidant defenses in the lung, even under pathological conditions. Although the elevation of SOD activity in trained IPF mice was not like that found in the Exe group (without fibrosis), the improvement of antioxidant enzymatic defenses sustains the putative benefit of exercise training on pulmonary fibrosis. In addition, a study from Du et al. (2019) has demonstrated that physical exercise can restore the synthesis of pulmonary hydrogen sulfide (H2S), improving the features of IPF in a model of pulmonary fibrosis induced by bleomycin [53]. This is of importance since H2S is a gasotransmitter similar to nitric oxide (NO), which has been increased in the exercised groups (Exe and Exe+Bleo), and according to the literature, increased levels of NO present protective effects in bleomycin model of pulmonary fibrosis [33, 37, 38]. Furthermore, a study from Prata et al. (2017) has additionally demonstrated that angiotensin-converting enzyme 2 (ACE2) is activated by physical exercise leading to reduction of pulmonary fibrosis [54].

In conclusion, our results showed, for the first time, that when aerobic exercise training is concomitantly initiated with pulmonary fibrosis induction by bleomycin, some physiological benefits were observed, such as significant reduction of both lung and systemic inflammation, improvement of the antioxidant capacity, and a hypothetical optimized oxygen uptake in lungs, although other mechanical parameters associated with lung alterations, such as impairment of fibrosis and lung mechanics, were not influenced by the exercise training.

## Data Availability

Answer: Yes. Comment.

## References

[1] Martinez F. J., Collard H. R., Pardo A. (2017). Idiopathic pulmonary fibrosis. *Nature Reviews. Disease Primers*.

[2] Nakamura Y., Suda T. (2015). Idiopathic pulmonary fibrosis: diagnosis and clinical manifestations. *Clinical Medicine Insights: Circulatory, Respiratory and Pulmonary Medicine*.

[3] Raghu G., Rochwerg B., Zhang Y. (2015). An Official ATS/ERS/JRS/ALAT Clinical Practice Guideline: treatment of idiopathic pulmonary fibrosis. An update of the 2011 Clinical Practice Guideline. *American Journal of Respiratory and Critical Care Medicine*.

[4] Dowman L., Hill C. J., Holland A. E. (2014). Pulmonary rehabilitation for interstitial lung disease. *Cochrane Database of Systematic Reviews*.

[5] da Fontoura F. F., Berton D. C., Watte G. (2018). Pulmonary rehabilitation in patients with advanced idiopathic pulmonary fibrosis referred for lung transplantation. *Journal of Cardiopulmonary Rehabilitation and Prevention*.

[6] Gomes-Neto M., Silva C. M., Ezequiel D., Conceição C. S., Saquetto M., Machado A. S. (2018). Impact of pulmonary rehabilitation on exercise tolerance and quality of life in patients with idiopathic pulmonary fibrosis. *Journal of Cardiopulmonary Rehabilitation and Prevention*.

[7] Dowman L. M., McDonald C. F., Hill C. J. (2017). The evidence of benefits of exercise training in interstitial lung disease: a randomised controlled trial. *Thorax*.

[8] Pereira P. R, Oliveira-Junior M. C, Mackenzie B. (2016). Exercise reduces lung fibrosis involving serotonin/Akt signaling. *Medicine and Science in Sports and Exercise*.

[9] Prata L. O., Oliveira F. M. S., Ribeiro T. M. S. (2012). Exercise attenuates pulmonary injury in mice with bleomycin-induced pulmonary fibrosis. *Experimental Biology and Medicine*.

[10] Mouratis M. A., Aidinis V. (2011). Modeling pulmonary fibrosis with bleomycin. *Current Opinion in Pulmonary Medicine*.

[11] Reis Gonçalves C. T., Reis Gonçalves C. G., de Almeida F. M. (2012). Protective effects of aerobic exercise on acute lung injury induced by LPS in mice. *Critical Care*.

[12] Toledo A. C., Magalhaes R. M., Hizume D. C. (2012). Aerobic exercise attenuates pulmonary injury induced by exposure to cigarette smoke. *The European Respiratory Journal*.

[13] Vieira R. P., Toledo A. C., Silva L. B. (2012). Anti-inflammatory Effects of Aerobic Exercise in Mice Exposed to Air Pollution. *Medicine & Science in Sports & Exercise*.

[14] Vieira R. P., Claudino R. C., Duarte A. C. S. (2007). Aerobic exercise decreases chronic allergic lung inflammation and airway remodeling in mice. *American Journal of Respiratory and Critical Care Medicine*.

[15] Pastva A., Estell K., Schoeb T. R., Atkinson T. P., Schwiebert L. M. (2004). Aerobic exercise attenuates airway inflammatory responses in a mouse model of atopic asthma. *Journal of Immunology*.

[16] Postal M., Appenzeller S. (2015). The importance of cytokines and autoantibodies in depression. *Autoimmunity Reviews*.

[17] Ramos D. S., Olivo C. R., Quirino Santos Lopes F. D. (2010). Low-intensity swimming training partially inhibits lipopolysaccharide-induced acute lung injury. *Medicine and Science in Sports and Exercise*.

[18] Patki G., Li L., Allam F. (2014). Moderate treadmill exercise rescues anxiety and depression-like behavior as well as memory impairment in a rat model of posttraumatic stress disorder. *Physiology & Behavior*.

[19] Durigon T. S., MacKenzie B. A., Oliveira-Junior M. C. (2018). Aerobic Exercise Protects from *Pseudomonas aeruginosa*-Induced Pneumonia in Elderly Mice. *Journal of Innate Immunity*.

[20] Kradin R. L., Sakamoto H., Jain F., Zhao L. H., Hymowitz G., Preffer F. (2004). IL-10 inhibits inflammation but does not affect fibrosis in the pulmonary response to bleomycin. *Experimental and Molecular Pathology*.

[21] Andrade-Sousa A. S., Pereira P. R., MacKenzie B. (2016). Aerobic Exercise Attenuated bleomycin-induced lung fibrosis in Th2-dominant mice. *PLoS One*.

[22] Alberca-Custódio R. W., Greiffo F. R., MacKenzie B. A. (2016). Aerobic exercise reduces asthma phenotype by modulation of the leukotriene pathway. *Frontiers in Immunology*.

[23] Hallstrand T. S., Lai Y., Henderson W. R., Altemeier W. A., Gelb M. H. (2012). Epithelial regulation of eicosanoid production in asthma. *Pulmonary Pharmacology & Therapeutics*.

[24] Luo M., Lee S., Brock T. G. (2003). Leukotriene synthesis by epithelial cells. *Histology and Histopathology*.

[25] PETERS-GOLDEN M. A. R. C., BAILIE M. A. R. C., MARSHALL T. E. R. E. S. A. (2002). Protection from pulmonary fibrosis in leukotriene-deficient mice. *American Journal of Respiratory and Critical Care Medicine*.

[26] Wilborn J., Bailie M., Coffey M., Burdick M., Strieter R., Peters-Golden M. (1996). Constitutive activation of 5-lipoxygenase in the lungs of patients with idiopathic pulmonary fibrosis. *The Journal of Clinical Investigation*.

[27] Izumo T., Kondo M., Nagai A. (2009). Effects of a leukotriene B4 receptor antagonist on bleomycin-induced pulmonary fibrosis. *European Respiratory Journal*.

[28] Nakagome K., Dohi M., Okunishi K., Tanaka R., Miyazaki J., Yamamoto K. (2006). In vivo IL-10 gene delivery attenuates bleomycin induced pulmonary fibrosis by inhibiting the production and activation of TGF-*β* in the lung. *Thorax*.

[29] Vannella K. M., Wynn T. A. (2017). Mechanisms of organ injury and repair by macrophages. *Annual Review of Physiology*.

[30] Mosser D. M., Edwards J. P. (2008). Exploring the full spectrum of macrophage activation. *Nature Reviews. Immunology*.

[31] Gong D., Shi W., Yi S. J., Chen H., Groffen J., Heisterkamp N. (2012). TGF*β* signaling plays a critical role in promoting alternative macrophage activation. *BMC Immunology*.

[32] Ip W. K. E., Hoshi N., Shouval D. S., Snapper S., Medzhitov R. (2017). Anti-inflammatory effect of IL-10 mediated by metabolic reprogramming of macrophages. *Science*.

[33] Nosarev A. V., Smagliy L. V., Anfinogenova Y., Popov S. V., Kapilevich L. V. (2015). Exercise and NO production: relevance and implications in the cardiopulmonary system. *Frontiers in Cell and Developmental Biology*.

[34] Rath M., Müller I., Kropf P., Closs E. I., Munder M. (2014). Metabolism via arginase or nitric oxide synthase: two competing arginine pathways in macrophages. *Frontiers in Immunology*.

[35] Farkas L., Gauldie J., Voelkel N. F., Kolb M. (2011). Pulmonary hypertension and idiopathic pulmonary fibrosis: a tale of angiogenesis, apoptosis, and growth factors. *American Journal of Respiratory Cell and Molecular Biology*.

[36] Duong-Quy S., Hua-Huy T., Pham H. (2014). Early inhaled nitric oxide at high dose enhances rat lung development after birth. *Nitric Oxide*.

[37] Noguchi S., Yatera K., Wang K. Y. (2014). Nitric oxide exerts protective effects against bleomycin-induced pulmonary fibrosis in mice. *Respiratory Research*.

[38] Thornadtsson A., Drca N., Ricciardolo F., Högman M. (2017). Increased levels of alveolar and airway exhaled nitric oxide in runners. *Upsala Journal of Medical Sciences*.

[39] Patel D., Lakhkar A., Wolin M. S. (2017). Redox mechanisms influencing cGMP signaling in pulmonary vascular physiology and pathophysiology. *Advances in Experimental Medicine and Biology*.

[40] Della Latta V., Cecchettini A., Del Ry S., Morales M. A. (2015). Bleomycin in the setting of lung fibrosis induction: from biological mechanisms to counteractions. *Pharmacological Research*.

[41] Saleh D., Barnes P. J., Giaid A. (1997). Increased production of the potent oxidant peroxynitrite in the lungs of patients with idiopathic pulmonary fibrosis. *American Journal of Respiratory and Critical Care Medicine*.

[42] He C., Ryan A. J., Murthy S., Carter A. B. (2013). Accelerated development of pulmonary fibrosis via Cu, Zn-superoxide dismutase-induced alternative activation of macrophages. *The Journal of Biological Chemistry*.

[43] Miura T. (2015). The peroxidase activity of bleomycin-Fe3+ is associated with damage to biological components. *Journal of Biochemistry*.

[44] Chow M. S., Liu L. V., Solomon E. I. (2008). Further insights into the mechanism of the reaction of activated bleomycin with DNA. *Proceedings of the National Academy of Sciences*.

[45] Allawzi A., Elajaili H., Redente E. F., Nozik-Grayck E. (2019). Oxidative toxicology of bleomycin: role of the extracellular redox environment. *Curr Opin Toxicol.*.

[46] Sies H., Jones D. P. (2020). Reactive oxygen species (ROS) as pleiotropic physiological signalling agents. *Nature Reviews. Molecular Cell Biology*.

[47] Nemmar A., Al-Salam S., Yuvaraju P., Beegam S., Ali B. H. (2018). Exercise training mitigates water pipe smoke exposure-induced pulmonary impairment via inhibiting NF-*κ*B and activating Nrf 2 signalling pathways. *Oxidative Medicine and Cellular Longevity*.

[48] Cho H. Y., Kleeberger S. R. (2015). Association of Nrf 2 with airway pathogenesis: lessons learned from genetic mouse models. *Archives of Toxicology*.

[49] Pall M. L., Levine S. (2015). Nrf 2, a master regulator of detoxification and also antioxidant, anti-inflammatory and other cytoprotective mechanisms, is raised by health-promoting factors. *Sheng Li Xue Bao*.

[50] Rahman A., Fazal F. (2011). Blocking NF-*κ*B: an inflammatory issue. *Proceedings of the American Thoracic Society*.

[51] da Cunha M. J., da Cunha A. A., Ferreira G. K. (2013). The effect of exercise on the oxidative stress induced by experimental lung injury. *Life Sciences*.

[52] Rigonato-Oliveira N. C., Mackenzie B., Bachi A. L. L. (2018). Aerobic exercise inhibits acute lung injury: from mouse to human evidence exercise reduced lung injury markers in mouse and in cells. *Exercise Immunology Review*.

[53] Du S. F., Wang X. L., Ye C. L. (2019). Exercise training ameliorates bleomycin-induced epithelial mesenchymal transition and lung fibrosis through restoration of H2S synthesis. *Acta Physiologica*.

[54] Prata L. O., Rodrigues C. R., Martins J. M. (2016). Original research: ACE2 activator associated with physical exercise potentiates the reduction of pulmonary fibrosis. *Experimental Biology and Medicine*.

